# Supplementary material for: Cytomegalovirus Blood DNAemia in Patients with Severe SARS-CoV-2 Pneumonia
Source: Infect Dis Rep. 2025 Jan 26;17(1):8. doi: 10.3390/idr17010008 (PMC11855763; doi:10.3390/idr17010008)
Supplement: Supplementary file 1 [file idr-17-00008-s001.zip › idr-3275247-supplementary.pdf]

Supplementary materials

Table S1. Characteristics and outcomes between patients tested or not for cytomegalovirus blood reactivation.

	Not tested (n = 53)	Tested (n = 135)	p value
Age, years	67 (58;73)	63 (55;69)	0.054
APACHE II	18 (15;23)	15 (12;18)	<0.0001
SOFA	8 (5 ;9)	6 (4;7)	0.0003
Admission CRP, mg/dL	131 (72;235)	142 (82;213)	0.83
PaO2/FiO2 (admission)	75 (59;97)	66 (53;82)	0.022
Hypertension (%)	38 (72)	75 (56)	0.042
Diabetes mellitus (%)	24 (46)	45 (33)	0.10
Cardiomyopathy (%)	23 (43)	26 (19)	0.0007
Neoplasia < 2 years (%)	12 (23)	9 (7)	0.0018
Immunosuppression (%)	11 (21)	25 (19)	0.74
COPD (%)	2 (4)	7 (5)	0.68
Chronic kidney disease (%)	12 (23)	12 (9)	0.011
ICU mortality (%)	36 (68)	88 (65)	0.52
Duration of ventilation, days	7 (4;14)	24 (14;56)	<0.0001
ICU length of stay, days	10 (6;22)	33 (19;69)	<0.0001

ICU: intensive care unit, COPD: chronic obstructive pulmonary disease, SOFA: Sequential Organ Failure Assessment, APACHE II: Acute Physiology and Chronic Health Evaluation II.

Table S2. Characteristics and outcomes between patients with absence of CMV DNAemia, CMV DNAemia < 500 IU/mL and CMV DNAemia > 500 IU/mL.

	Absence of CMV DNAemia (n = 52)	CMV DNAemia < 500 UI/mL (n = 53)	CMV DNAemia > 500 UI/mL (n = 30)	p value
Age, years	63 (54 ;68)	63 (55 ;72)	63 (55;69)	0.89
APACHE II	15 (13;18)	16 (12;19)	14 (12 ;19)	0.63
SOFA	8 (5 ;9)	5 (4;8)	6 (5 ;8)	0.83
BMI	28 (26 ;31)	28 (24,33)	30 (26 ;32)	0.97
PaO2/FiO2 (admission)	62 (50 ;78)	71 (56 ;91)	64 (54 ;78)	0.23
Lowest PaO2/FiO2	47 (41 ;51)	48 (43 ;52)	49 (45 ;53)	0.58
Hypertension (%)	25 (48)	29 (55)	21 (70)	0.16
Diabetes mellitus (%)	21 (40)	16 (30)	8 (27)	0.37
Cardiomyopathy (%)	10 (19)	12 (23)	4 (13)	0.59
Neoplasia < 2 years (%)	6 (12)	3 (6)	0 (0)	0.13
Immunosuppression (%)	12 (23)	8 (15)	6 (20)	0.58
COPD (%)	5 (10)	2 (4)	0 (0)	0.14
Chronic kidney disease (%)	6 (12)	4 (8)	2 (7)	0.69
Dexamethasone (%)	37 (71)	40 (75)	28 (93)	0.047
ECMO (%)	15 (29)	14 (26)	11 (37)	0.61
ICU mortality (%)	37 (71)	34 (64)	17 (57)	0.56
Duration of ventilation, days	17 (9;37)	23 (16 ;44)	63 (41 ;83)	<0.0001
ICU length of stay, days	22 (12 ;38)	29 (20 ;57)	68 (48 ;97)	<0.0001

ICU: intensive care unit, COPD: chronic obstructive pulmonary disease, SOFA: Sequential Organ Failure Assessment, APACHE II: Acute Physiology and Chronic Health Evaluation II.

Table S3. Characteristics and outcomes between patients with CMV DNAemia > 500 IU/mL treated or not with valganciclovir.

	**No valganciclovir (n = 14)**	**Valganciclovir (n = 16)**	**p value**
Age, years	66 (62;75)	57 (51;65)	0.0061
APACHE II	16 (13;19)	12 (10;18)	0.036
SOFA	6 (5;7)	6 (5;8)	0.76
BMI	28 (26;31)	31 (25;36)	0.27
PaO2/FiO2 (admission)	63 (56;79)	64 (47;78)	0.71
Lowest PaO2/FiO2	49 (43;53)	50 (46;54)	0.73
CMV DNA (IU/mL)	1759 (839;4419)	6293 (2831;8029)	0.0060
Hypertension (%)	10 (71)	11 (69)	>0.99
Diabetes mellitus (%)	4 (29)	4 (25)	>0.99
Cardiomyopathy (%)	2 (14)	2 (13)	>0.99
Neoplasia < 2 years (%)	0 (0)	0 (0)	>0.99
Immunosuppression (%)	1 (7)	5 (31)	0.18
COPD (%)	0 (0)	0 (0)	>0.99
Chronic kidney disease (%)	0 (0)	2 (13)	0.49
Dexamethasone (%)	12 (86)	16(100)	0.12
ECMO (%)	1 (7)	10 (63)	0.0017
ICU mortality (%)	8 (57)	9 (56)	>0.99
Duration of ventilation, days	54 (38;72)	72 (49;102)	0.13
ICU length of stay, days	63 (42;80)	80 (55;109)	0.20

ICU: intensive care unit, COPD: chronic obstructive pulmonary disease, SOFA: Sequential Organ Failure Assessment, APACHE II: Acute Physiology and Chronic Health Evaluation II.
